# The Validity of the Hospital Anxiety and Depression Scale and the Geriatric Depression Scale in Parkinson’s Disease

**DOI:** 10.1155/2006/136945

**Published:** 2006-07-17

**Authors:** Federica Mondolo, Marjan Jahanshahi, Alessia Granà, Emanuele Biasutti, Emanuela Cacciatori, Paolo Di Benedetto

**Affiliations:** ^1^Institute of Physical Medicine and RehabilitationGervasutta HospitalVia Gervasutta4833100 UdineItaly; ^2^Sobell Department of Motor Neuroscience & Movement DisordersInstitute of NeurologyThe National Hospital for Neurology & NeurosurgeryQueen SquareLondon WC1N 3BGUK; ^3^Department of PsychologyUniversity of TriesteItaly

**Keywords:** Parkinson’s disease, depression, Hospital Anxiety and Depression Scale (HADS), Geriatric Depression Scale (GDS)

## Abstract

We assessed the concurrent validity of the Hospital Anxiety and Depression Scale (HADS) and the Geriatric Depression Scale (GDS) against the Hamilton Rating Scale for Depression (Ham-D) in patients with Parkinson’ disease (PD). Forty-six non-demented PD patients were assessed by a neurologist on the Ham-D. Patients also completed four mood rating scales: the HADS, the GDS, the VAS and the Face Scale. For the HADS and the GDS, Receiver Operating Characteristics (ROC) curves were obtained and the positive and negative predictive values (PPV, NPV) were calculated for different cut-off scores. Maximum discrimination between depressed and non-depressed PD patients was reached at a cut-off score of 10/11 for both the HADS and the GDS. At the same cut-off score of 10/11 for both the HADS and the GDS, the high sensitivity and NPV make these scales appropriate screening instruments for depression in PD. A high specificity and PPV, which is necessary for a diagnostic test, was reached at a cut-off score of 12/13 for the GDS and at a cut-off score of 11/12 for the HADS. The results indicate the validity of using the HADS and the GDS to screen for depressive symptoms and to diagnose depressive illness in PD.

## 1. Introduction

Depression is the most frequent psychiatric disorder in patients with Parkinson's disease (PD) [1,2].

Previous studies have indicated that the frequency of depression in PD is about 40%, with reported rates ranging from 4% to 70% [3,4]. The two main variables that account for the discrepancies in the prevalence of depression in PD are: sampling methodology (community vs. hospital-based samples) and the case ascertainment criteria (cut-off scores on depression scales vs. standardized diagnostic criteria based on semi-structured psychiatric interviews). Previous studies with high rates of depression in PD have been based mostly on selected hospital-based patient samples or have relied on rating scales [5–8].

Diagnosis of depression in a patient with PD is a critical clinical problem. In fact, it is difficult to obtain a valid diagnosis of depression in patients with a neurological illness, because the neurological disease itself, independently of the depressive disorder, may produce symptoms that overlap with those that are central to the diagnosis of mood disorders. Patients with PD and patients with "primary" depressive illness may show symptoms such as bradykinesia, motor retardation, a blank facial expression, apathy, a stooped posture, and sleeping problems. For this reason, the use of depression rating scales for assessing depression in parkinso-

ISSN 0953-4180/06/$17.00 © 2006 – IOS Press and the authors. All rights reserved nian patients is often criticized because the inclusion of somatic items in these scales may make it difficult to differentiate between the depressive symptoms and the motor symptoms of PD. Nevertheless, in light of their practical utility and ease of administration, use of rating scales for the evaluation of depression in PD can not be dismissed. Consequently, the validity of the Hamilton Rating Scale for Depression (Ham-D), the Montgomery-Asberg Depression Scale, and the Beck Depression Inventory for specific use in PD has been evaluated, and cut-off scores for using the scales for screening or diagnosis of depression in PD have been established in a number of studies [9–12].

The primary objective of the present study was to examine the concurrent validity of two other depression scales commonly used in PD, the Hospital Anxiety and Depression Scale (HADS) [13] and the Geriatric Depression Scale (GDS) [14], for assessing the severity of depressive symptoms in patients with Parkinson's disease (PD). To establish the concurrent validity of these two depression scales, we used the Ham-D [15] as the "gold standard" instead of the DSM-IV diagnostic criteria for depressive illness, because of the proven high sensitivity and specificity of the Ham-D for PD patients in recent studies [9,10]. Our second aim was to evaluate the utility of brief non-verbal methods such as the Face Scale [16], and a Visual Analogue Scale for depression (VAS) [17] to reliably and validly assess depressed mood in PD patients.

## 2. Methods

### 2.1. Sample

Forty-six patients with PD (28 males and 18 females), diagnosed according to the clinical criteria of the United Kingdom Parkinson's Disease Society Brain Bank (UK-PDS-BB) [18] participated in the study. The patients were consecutive referrals from the Department of Physical Medicine of the "Gervasutta" Rehabilitation Hospital, Udine, for a standardized 'mental status' examination.

Patients were screened for dementia using the Mini Mental State Examination (MMSE) [19] and those with a score below 24 were excluded. Severity of PD was rated according to the Hoehn and Yahr stage of illness scale in the 'on' medication state [20]. Patients with atypical parkinsonism, vascular parkinsonism, drug-induced parkinsonism, and those with parkinsonism following dementia were excluded. The majority of the 46 patients were taking dopamine receptor agonists (36 patients) and/or L-dopa therapy (40 patients). Demographic and clinical features of the PD patients are summarized in Table 1.

Table 1
Demographic and clinical features of the PD patients ($n = 46$)

| | Means | SD |
|---|---|---|
| Age (years) | 67.7 | 8.2 |
| Sex (female/male) | 18 (39) | |
| Age at Onset (years) | 62.6 | 9.9 |
| Duration of illness (years) | 6.0 | 4.0 |
| Positive family history | 9 (19.6) | |
| Hoehn and Yahr score | 2.1 | 0.5 |
| MMSE | 27.8 | 1.5 |
| Education (years) | 7.8 | 3.8 |

MMSE, Mini Mental State Examination Values in parentheses are %.

### 2.2. Procedure

The 'mental state' examination consisted of a semi-structured interview with a neurologist using the Hamilton Rating Scale for Depression (Ham-D) [15], for the diagnosis of depressive disorder. The Ham-D diagnosis of depressive disorder was considered the 'gold standard' for depression in this study. All patients were asked to complete the Geriatric Depression Scale (GDS) [14], the Hospital Anxiety and Depression Scale (HADS) [13], a Visual Analogue Scale [17] and a Face Scale [16].

All patients completed the depression rating scales during two different testing sessions with a psychologist, with a gap of one week between the two sessions. In the first session, patients completed the Geriatric Depression Scale (GDS) [14]. In the second session they were asked to complete the Hospital Anxiety and Depression Scale (HADS) [13], a Visual Analogue Scale [17] and a Face Scale [16]. At the end of the second testing session, there was a semi-structured interview with a neurologist using the Hamilton Depression Rating Scale (Ham-D) [15].

The Hoehn & Yahr staging of illness [20] was completed by a neurologist during a semi-structured clinical interview and neurological examination. Details of the patients' medical history were extracted from their general practitioners' records.

### 2.3. Depression rating scales

The GDS consists of twenty items, higher scores (range 0–30) indicate more severe depression. The HADS consists of seven depression items and seven

Table 2
Spearman correlation coefficients between the different depression scales

| | HADS | GDS | HAM-D | Face scale | VAS depression |
|---|---|---|---|---|---|
| HADS | | $r = 0.72^{**}$ | $r = 0.61^{**}$ | $r = 0.52^{**}$ | $r = -0.64^{**}$ |
| GDS | | | $r = 0.59^{**}$ | $r = 0.39^{**}$ | $r = -0.47^{**}$ |
| HAM-D | | | | $r = 0.48^{**}$ | $r = -0.31^{*}$ |
| Face Scale | | | | | $r = -0.57^{**}$ |
| VAS | | | | | |

$^{**}$Correlation is significant at the 0.01 level (2-tailed).
$^{*}$Correlation is significant at the 0.05 level (2-tailed).

anxiety items. In this study we consider only the depression subscale. All items are rated on a four-point scale, ranging from the absence of a symptom (score of 0) to maximum symptomatology (score of 3). On the depression subscale of the HADS, higher scores (range 0–21) indicate more severe depression. The Ham-D scale contains 17 items, which are scored either on five-point or three-point scales. Higher scores indicate more severe depression.

The VAS consists of a 10-cm vertical line without subdivisions or numbers, anchored at one end by a smiling face, representing the most positive mood (max score of 10) and at the other end by a sad face, representing the most negative mood (lowest score of 0). The Face Scale contains 20 drawings of a single face, arranged in serial order in rows, with each face depicting a slightly different mood state. The faces are arranged in increasing order of mood and numbered from 1–20, with 1 representing the most positive mood and 20 representing the most negative mood.

### *2.4. Analysis*

For each scale, scores were calculated according to the respective scoring algorithms. In order to determine the sensitivity and specificity of the HADS and the GDS scales as screening and diagnostic tools for depression in PD, against the 'gold standard' provided by the Ham-D and to obtain the optimal cut-off points, "receiver operating characteristics" curves (ROC curve) [21] were plotted for each scale. For statistical analysis, we selected non-parametric tests because of the ordinal nature of the scales. We analysed associations between scales with Spearman rank correlations. All statistical analyses were performed with SPSS for Windows [22].

## 3. Results

Demographic and clinical features of the PD patients are summarized in Table 1.

On the Ham-D, the mean scores for the 46 patients was 5.30 (SD 3.53; range 0–13). On the HADS and the GDS, the average scores for the sample were respectively 7.09 (SD 3.32; range 1–13) and 9.17 (SD 5.34; range 1–24). Five patients met the Ham-D criteria for depressive disorder, corresponding to a rate of 11%. Two patients met the GDS criteria for severe depression (score $\geqslant$ 20), 16 met the criteria for mild depression (score $\geqslant$ 10) and 28 were non-depressed. Seven patients met the HADS criteria for severe depression (score $\geqslant$ 11), 11 met the criteria for mild depression (score $\geqslant$ 8) and 28 were non-depressed.

We compared the results on all three depression scales administered for patients who were or were not taking dopaminergic drugs. There were no significant differences on the Ham-D, the HADS or the GDS between patients taking dopamine receptor agonists and patients who did not take them. Similarly, there was there was no significant differences on the Ham-D, the HADS or the GDS between patients taking or not taking L-dopa.

### *3.1. Correlational analysis*

The Spearman correlation coefficients between the different depression scales are presented in Table 2.

There was a good correlation between the different depression rating scales, with the magnitude of the correlations ranging from 0.39 to 0.72 (all $p < 0.01$; see Table 2), and there was a trend for an association between the Ham-D and the VAS depression ($r = -0.31$; $p < 0.05$).

### *3.2. Receiver operating characteristics curve*

Sensitivity, specificity, positive and negative predictive values for different cut-off scores are shown in Table 3 for the GDS and in Table 4 for the HADS. Figures 1 and 2 display the ROC curve for the HADS and GDS, respectively. Maximal discrimination between non-depressed and depressed PD patients is reached at

Table 3
Sensitivity, specificity, positive and negative predictive values at different cut-off scores for the Geriatric Depression Scale

| Cut-off | Sensitivity | Specificity | PPV | NPV |
|---|---|---|---|---|
| 7/8 | 1.00 | 0.39 | 0.17 | 1.00 |
| 8/9 | 1.00 | 0.49 | 0.19 | 1.00 |
| 9/10 | 1.00 | 0.68 | 0.28 | 1.00 |
| 10/11* | 1.00 | 0.76 | 0.33 | 1.00 |
| 11/12 | 0.80 | 0.80 | 0.33 | 0.97 |
| 12/13 | 0.80 | 0.85 | 0.40 | 0.97 |
| 13/14 | 0.60 | 0.85 | 0.33 | 0.94 |

PPV, positive predictive value.
NPV, negative predictive value.
*Maximum sum of sensitivity and specificity.

Table 4
Sensitivity, specificity, positive and negative predictive values at different cut-off scores for the Hospital Anxiety and Depression Scale

| Cut-off | Sensitivity | Specificity | PPV | NPV |
|---|---|---|---|---|
| 9/10 | 1.00 | 0.76 | 0.33 | 1.00 |
| 10/11* | 1.00 | 0.95 | 0.71 | 1.00 |
| 11/12 | 0.80 | 0.98 | 0.80 | 0.98 |
| 12/13 | 0.40 | 0.98 | 0.66 | 0.93 |
| 13/14 | 0.00 | 1.00 | 0.00 | 0.89 |

PPV, positive predictive value.
NPV, negative predictive value.
*Maximum sum of sensitivity and specificity.

the cut-off point that has the highest sum of sensitivity and specificity. This point can be determined visually from the ROC curve. For the HADS, this optimal cut-off score is 10/11 (sensitivity 1.00, specificity 0.95), meaning that a score of 10 or less indicates the absence of depression and a score of 11 or higher is indicative of the presence of depression. For the GDS, the optimal cut-off score is also 10/11 (sensitivity 1.00, specificity 0.76), meaning that a score of 10 or less indicates the absence of depression and a score of 11 or higher is indicative of the presence of depression. The area under the curve (AUC) were high for both scales, 0.895 for the GDS ($z = 4{,}11$; $p < 0.05$) and 0.978 for the HADS ($z = 9.56$; $p < 0.05$). The larger AUC for the HADS indicated that this scale was better than the GDS for diagnosing depression in PD patients. Cut-off values can be set depending on the purpose for which the scales are used. For screening purposes, a high sensitivity and a high NPV are required. At a cut-off score of 10/11 for both the GDS and the HADS, these requirements were fulfilled. At this cut-off score, the sensitivity and the NPV were the same for both scales. For diagnostic purposes a high specificity and a high positive predictive value are required. Cut-off scores of 12/13 for the GDS and of 11/12 for the HADS increased their specificity and PPV. At these cut-off scores, both specificity and PPV of the HADS were higher than those of the GDS.

## 4. Discussion

The Hamilton depression scale is one of the most frequently used clinician-rated depression symptom severity scales. However, in practice, the Ham-D is not always practical to use because its completion requires too much time and presence of a trained clinician. Self-report questionnaires represent a practical option for objectively evaluating mood in PD patients. For example, the two scales that we used in this study, the HADS and the GDS, require no more than 5 and 8 minutes respectively and patients find them easy to complete. However, reliance on self-rating scales for depression raises the problem of the "validity" of the patients' answers, which could be biased by factors such as reporting bias, lack of awareness of the symptoms and cognitive impairment.

We evaluated whether the HADS or GDS can be validly used as screening and diagnostic scales for depression in PD. We used the Ham-D as the "gold standard" for depressive illness, because of the proven high sensitivity and specificity of the Ham-D for PD patients in recent studies [9,10]. In particular, to dichotomize the population into depressed versus non-depressed, we used the screening cut-off score for Ham-D proposed by Leentjens et al. [9] of 11/12. We determined the concurrent validity of the HADS and GDS against the Ham-D. We found that at a cut-off score of 10/11 for both the HADS and the GDS, these two scales can be used as screening tools for depression, because both questionnaires show a high sensitivity and a high negative predictive value. Increasing the cut-off score to 12/13 for the GDS and 11/12 for the HADS, these scales also proved to be useful as diagnostic tools. While the HADS and the GDS were not designed as diagnostic scales, at these cut-off scores both scales show a high specificity and a high positive predictive value. These results indicate that a single cut-off score for both screening and diagnostic purposes is not suitable for evaluating whether patients with PD are depressed or not. There was a significant association between self-rated depression on the HADS and GDS, and depression as evaluated by a neurologist on the Ham-D. The results further indicate that for PD patients, the self-ratings of depression on the HADS or GDS are valid indices of their mood.

Most self-rating scales for depression were developed for use in psychiatric populations and include somatic symptoms of depression such as motor retarda-

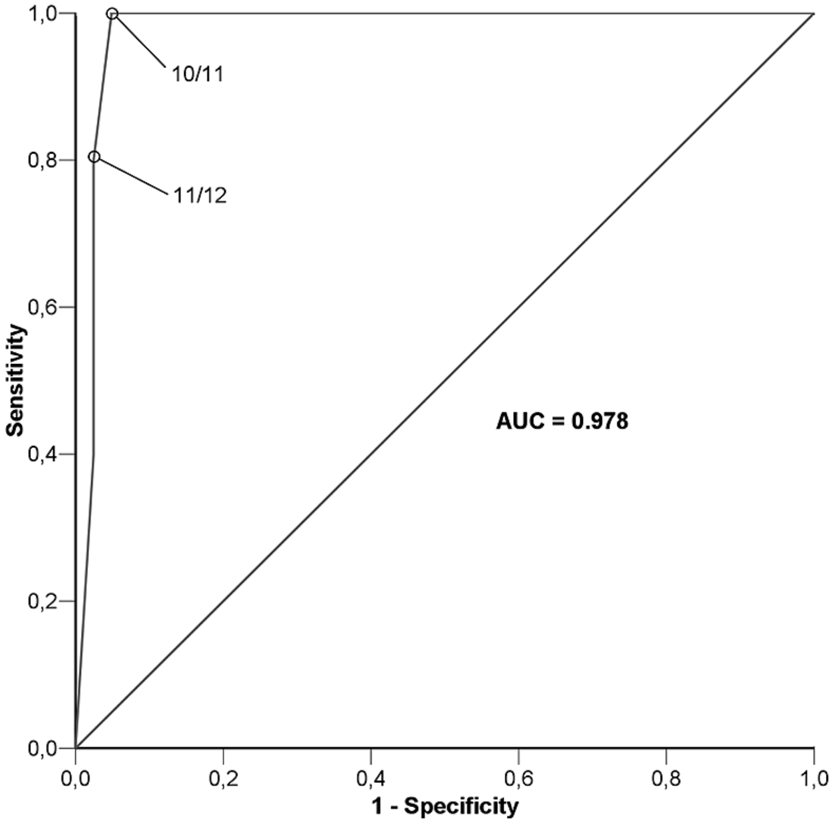

Fig. 1. Receiver operating characteristics (ROC) curve for the HADS. AUC = Area Under Curve.

tion, lack of energy and fatigability. Because these somatic features of depression show considerable overlap with those of PD, the prevalence of these symptoms may be overestimated when such scales are used in this population. In this respect the HADS and the GDS, are very suitable for use in PD because they do not have somatic items. Despite this obvious advantage, the GDS has hardly been used in this patient group. Recently Leentjens and colleagues [23] assessed the screening and diagnostic properties of the HADS and they concluded that the screening properties of HADS seem adequate, but that the diagnosis of depression is better achieved with expert-administered depression scales, such as the Ham-D and the MADRS [9]. More recently Marinus et al. [24], evaluated the psychometric properties of the HADS in a large population of patients with PD and they found that the reliability and construct validity of this scale was adequate. These authors also noted that, although the screening properties of the HADS seem adequate, the diagnostic properties may be questioned. Previous studies reported the concurrent validity of the Ham-D and the MADRS [9, 10], and of the BDI [12] against DSM-IV criteria for depressive disorder in patient with PD. In particular, the Ham-D showed the highest sum of sensitivity and specificity for both screening and diagnostic criteria. For this reason, we decided to use the Ham-D as the "gold standard" in our study. We found that the HADS and the GDS against the Ham-D criteria for depressive disorder are very good self-rating scales for evaluating mood in PD patients, because they have a high sensitivity and specificity, both for diagnostic and screening purposes.

One indication of a test's validity is its pattern of correlation with other established measures of the same construct. The correlations between the Face Scale and the VAS rating of depression with other standardized measures of mood were all statistically significant ($p < 0.01$; see Table 3). This indicates convergent validity of these scales. These results provide support for the use of the Face Scale and the VAS for depression as brief, non-verbal and valid methods for assessing mood in PD. The Face Scale and the VAS for rating depression were easily completed by patients with a minimal amount of guidance and required two minutes or less for completion. The Face Scale and the VAS for de-

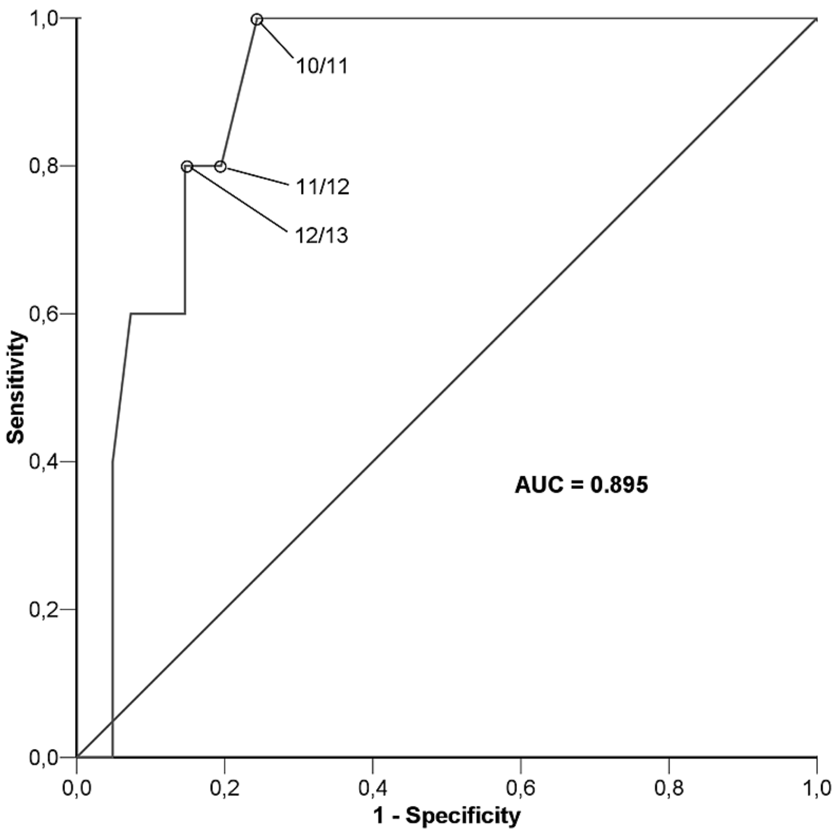

Fig. 2. Receiver operating characteristics (ROC) curve for the GDS. AUC = Area Under Curve.

pression are not intended to be used for the diagnosis of clinical depression, but they can be useful as screening tools prior to more extensive evaluation. Previous studies have also noted the value of the Face Scale in arthritis patients [16].

In conclusion, the concurrent validity of the HADS and the GDS against the Ham-D is high in PD. Maximum discrimination between non-depressed and depressed patients is reached at the cut-off score with the highest sum of sensitivity and specificity. This optimal cut-off score is 10/11 for both the GDS and the HADS. At the same cut-off score, the high sensitivity and NPV make these scales good screening instruments for depression in PD. A high specificity and PPV, which is necessary for a diagnostic test, was reached at a cut-off score of 11/12 for the HADS and 12/13 for the GDS. Thus a single cut-off score is not suitable for both screening and diagnosis of depression in PD.

Reliance on both, the HADS and the GDS, to measure depressive symptoms in PD patients and to diagnose depressive disorders in PD is appropriate. The Face Scale and the VAS for depression were shown to be brief, non-verbal screening tools for assessing mood in patients with PD.

## Acknoweledgments

This study was supported by the Association of Rehabilitation of Friuli Venezia Giulia (ART).
